# Optimization of the Rubber Formulation for Footwear Applications from the Response Surface Method

**DOI:** 10.3390/polym12092032

**Published:** 2020-09-07

**Authors:** Satta Srewaradachpisal, Charoenyutr Dechwayukul, Surapong Chatpun, Richard J. Spontak, Wiriya Thongruang

**Affiliations:** 1 Department of Mechanical Engineering, Faculty of Engineering, Prince of Songkla University, Hat Yai, Songkhla 90112, Thailand; satta.sre@gmail.com (S.S.); charoenyut.d@psu.ac.th (C.D.); 2 Institute of Biomedical Engineering, Faculty of Medicine, Prince of Songkla University, Hat Yai, Songkhla 90112, Thailand; schatpun@gmail.com; 3 Departments of Chemical & Biomolecular Engineering and Materials Science and Engineering, North Carolina State University, Raleigh, NC 27695, USA; spontak@ncsu.edu

**Keywords:** natural rubber, impact test, elastomer nanocomposite, RSM, footwear

## Abstract

Impact force remains the primary cause of foot injury and general discomfort with regard to footwear. The footwear industry traditionally relies on modified elastomers (including natural rubber) whose properties can be physically adjusted by varying the constituents in the rubber formulations. This work aims to investigate the effect of filler/plasticizer fractions on shock attenuation of natural rubber soles. The statistical response surface method (RSM) was used to optimize the loading of natural rubber, fillers (carbon black and china clay) and a plasticizer (paraffinic oil). A novel predictive equation addressing the effects of additives on the physical and mechanical properties of the shoe sole was successfully created using the RSM. Our results demonstrate how the concentrations of these components regulate final properties, such as impact force absorption and hardness, in the commercial manufacture of shoe soles. While a higher loading level of plasticizer promotes reductions in hardness and impact force, as well as energy dissipation, in these modified elastomers, these properties were improved by increasing the filler content.

## 1. Introduction

Impact force constitutes the leading cause of foot injury, with pain and discomfort in both feet and legs resulting from long periods of walking or, even worse, running [1,2,3,4]. Previous studies have highlighted health problems, such as bone fractures, cartilage degeneration, and osteoarthritis, as well as chronic knee and back pain. During running in particular, the impact load is typically increased to 1.5–5.0 × body weight [1,2,3,4,5]. The insertion of a cushion material in shoes can serve to protect the soft tissue on the human heel, along with the muscles in the legs and feet, and thus prevent improper body movement. Reducing the impact load can successfully prevent injury while increasing comfort when walking or running, thereby yielding a higher quality of life [2,4,6]. Presently, various foam materials derived from synthetic polymers, such as ethylene-vinyl acetate or polyurethane, are routinely employed in footwear products due to their well-known ability to absorb impact force. However, a nontrivial drawback of such foam materials is their relative lack of durability [2,7,8,9,10,11]. Therefore, to improve the durability of shoes and retain comfort, parts of many shoes are constructed from both natural rubber (NR) and synthetic materials. Previous studies of NR shoe soles in particular have focused on different elastomer considerations that are intended to reduce impact force. While products with a high NR content have been reported [2] to lower impact force, these studies have neglected considering the accompanying role of different additives, including their loading levels, incorporated into NR. Due to its desirable combination of elasticity and durability, NR is also widely used in cushioning and shoe applications.

A plethora of investigations has aimed at developing NR with designer properties, mostly in connection with the tire industry, by physically adjusting the content of fillers and oil. Increasing the content of carbon black (CB), for example, has been suggested as a means by which to improve the tensile properties, which are frequently related to hardness, of finished rubber, in contrast to increasing the content of a plasticizing oil [12,13,14]. Fillers and plasticizers have also been found to affect the internal energy loss, or equivalently the loss tangent, of elastomers due to internal friction (heat) generated by the material during compression [15,16,17,18]. The loss tangent correlates with the percentage resilience (PR) of an elastomer; that is, a higher loss tangent corresponds to a greater loss of energy and, in footwear, less force being transferred to the body [19]. A rebound pendulum can be used to measure PR, which is inversely proportional to the hysteresis loss or energy absorption. While such a device could be used to elucidate the systematic effect of varying the fraction of additives in NR products on relevant properties such as hardness, energy loss, and impact force, the necessary experimental matrix becomes unmanageably large. In this work, NR shoe soles containing different loading levels of two nanoscale fillers and one plasticizer are examined in a design of experiment (DoE) by using the RSM in conjunction with a quadratic regression model. The RSM examines the relationship between properties of interest and one or more response variables. Therefore, this work is novel in that it uses the RSM method to predict the effects of additives on physical and mechanical properties, focusing on the cushioning attributes of rubber. Moreover, we conduct a series of hardness measurements under the presumption that rubber hardness relates directly to peak impact force instead of using a rebound pendulum. The RSM approach adopted in this study enables a fewer number of experiments while all important experimental factors remain considered.

The objective here is to identify the optimal loading level of three commercial additives that are used together to generate a formulation exhibiting the greatest reduction in impact force. The DoE method employed in this work will be useful in future testing and design efforts, and our findings are anticipated to be of widespread interest to developers and manufacturers of footwear, especially with regard to shoes with soles that are produced from modified NR.

## 2. Materials and Methods

### 2.1. Materials

The NR (STR5L) rubber was purchased in block form from Chana Latex Co., Ltd. (Songkhla, Thailand), whereas CB (N-330, particles size 30 nm) and china clay (CC, C-400, mesh size 400) were acquired from GSP Products Co., Ltd. (Bangkok, Thailand). The CB and CC are generally considered as reinforcement and non-reinforcement fillers, respectively. Paraffinic oil (PO, grade A no.15), added to NR as a plasticizer to facilitate filler dispersion, was likewise obtained from GSP Products Co., Ltd. (Bangkok, Thailand). Other crosslinking species (zinc oxide, stearic acid, Wingstay L, 2-mercaptobenzothiazole (MBT), and sulfur) were all provided by Kij Paiboon Chemical Co., Ltd. (Bangkok, Thailand). Table 1 lists the compound formulations examined here.

Compound mixing was performed in two stages. First, the NR was masticated in a kneading machine (YFM Dispersion mixers 3L, Yong Fong Machinery Co., Ltd., Samutsakorn, Thailand) at 90 °C, at a roller speed of 30 rpm. Subsequently, the plasticizer and fillers were added according to the formulation in Table 1 within a mixing time of 30 min. Second, the modified NR was mixed with the remaining ZnO, Stearic acid and Wingstay L on a two-roll mill (YFM 400B, Yong Fong Machinery Co., Ltd., Samutsakorn, Thailand). After the compound was cooled to ambient temperature, it was mixed with MBT and sulfur and held at that temperature for 24 h to allow stress relaxation to proceed before vulcanization. The optimal curing time was chosen on the basis of prior reports [13,14,17] and in-house NR experience. Each test piece was molded at the same conditions of 150 °C and 150 kg/cm^2^ for 10–15 min and subsequently cooled at ambient temperature for 24 h prior to testing.

### 2.2. Experimental Design

Experimental design is a well-known procedure for testing hypotheses aiming to describe and possibly predict an outcome that is non-linearly dependent on multivariable conditions. The RSM algorithm used here creates a mathematical model to (*i*) discern the relationships that exist between experimental inputs and (*ii*) optimize designs that involve multiple variables by revealing the design response to independent changes in the variables. This DoE strategy greatly reduces the number of experiments necessary in a systematic (or full-factor) analysis. The independent variables examined here, as well as their ranges of interest, are included in Table 1. The DoE was conducted at three levels (low, mid, and high) of the three variables (CB, PO, and CC) to create a quadratic model given by

Y = a_0_ + a_1_X_1_ + a_2_X_2_ + a_3_X_3_ + a_4_X_1_^2^ + a_5_X_2_^2^ + a_6_X_3_^2^ + a_7_X_1_X_2_ + a_8_X_1_X_3_ + a_9_X_2_X_3_ (1)

where Y is the response variable, a0–a9 represent the values of the regression coefficients, and X_1_, X_2_, and X_3_ constitute the variables that are identified in Table 1. The data were analyzed with the Minitab 18 software package, and the results were derived from 20 experiments, with five replicates tested at the center point. Table 2 provides the experimental design matrix used in this study.

### 2.3. Testing Methods

**Figure 1:**
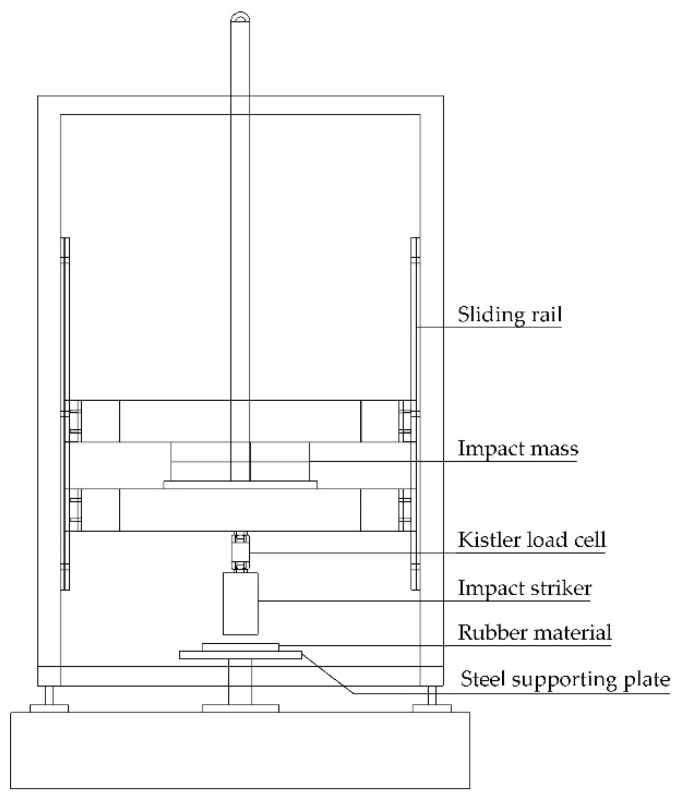
Illustration of the custom-built drop-testing machine used to measure impact energy.

The hardness of the vulcanized rubber compounds was measured with a durometer on a Shore A scale (Bareiss digi test II) according to ASTM D2240 at three different points on each specimen. The rebound PR from impact was also determined using the same instrument according to ASTM D1054. Shock absorption was evaluated according to ASTM F1614 on a custom-built drop-testing machine, as schematically depicted in Figure 1. The thickness of the molded rubber specimens to be tested was held constant at 10 mm. A striker with a mass of 8.5 kg and diameter of 45 mm was released from a height of 36–84 mm (depending on the striking energy required) onto each specimen, and the impact energy consequently generated varied from 3 to 7 J [20,21,22]. The impact force and the drop time were both measured by a 10 kN piezoelectric load cell (Kistler 9321b) and a data acquisition board (National Instruments USB-6008). The maximum reaction force returning from the first rebound was also recorded.

## 3. Results and Discussion

### 3.1. Statistical Analysis

Statistical analysis is widely used to study the trend and interpretation of results from testing data. As stated in the previous section, a statistical analysis of the results obtained here from the RSM-designed experiments was conducted in conjunction with the Minitab software, and statistical significance was judged at the 95% confidence level. Predicted property responses of the three variables identified in Equation (1) are listed in Table 3. Because the correlation (R^2^) values are all close to unity, the predictions are consistent with the experimental results. Adjusted R^2^ values compare the explanatory power of the regression models that contain different numbers of predictors. These are modifications of R^2^ to account for the number of predictors in the model and are generally less than R^2^. In this study, the adjusted R^2^ values of each model are relatively close to the value of R^2^, which confirms that the model equations developed according to the RSM are appropriate. Lastly, the predicted R^2^ constitutes a good indicator of how well the model can predict a new (i.e., untested) response. These values are also relatively high and not far from the adjusted R^2^ values, indicating that the models are also able to yield accurate property estimations. The equations that are presented in Table 3 display the mathematical relationships between linear and quadratic models representing the effects of the independent variables. The coefficients in the models signify the importance of the different variables in terms of linear, squared, and second-order correlations.

### 3.2. Property Correlations

The dependence of hardness on filler (CB and CC) and plasticizer (PO) loading level is linear, according to the model results that are shown in Table 3. This relationship is generated by analyzing experimental data in conjunction with the regression model in Equation (1) and applying the least-squares method for estimating the regression coefficients. According to the magnitude of the coefficients, the PO content (from the first order term X2) has the greatest influence in reducing hardness, whereas the addition of either filler (X1 for CB and X3 for CC) promotes an increase in hardness.

**Figure 2:**
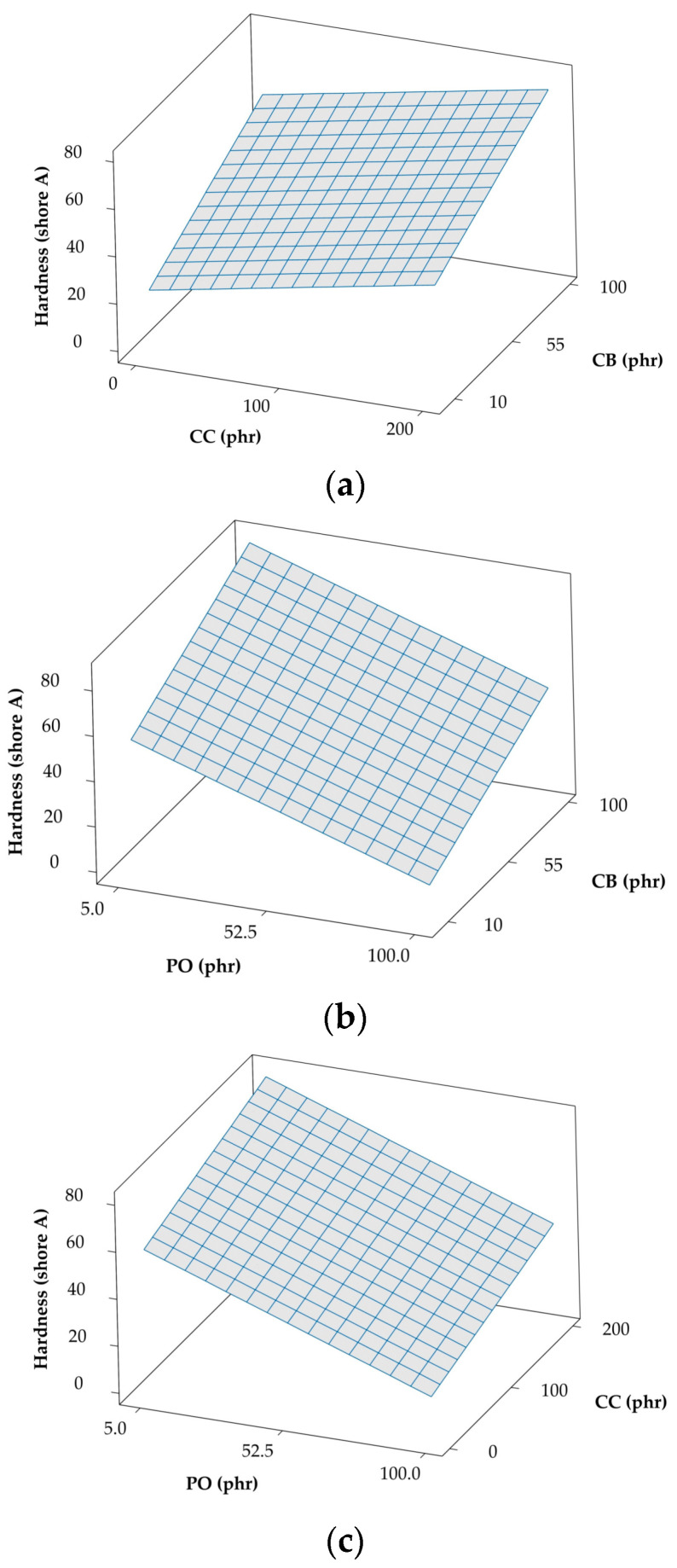
Response cross-correlation surface plots of hardness (Shore A) with various combinations of additives: (**a**) china clay (CC) and carbon black (CB) (paraffinic oil (PO) held constant at 52.5), (**b**) PO and CB (CC held constant at 100), and (**c**) PO and CC (CB held constant at 55).

The corresponding cross-correlation surface plots of hardness with two of these additives at a time are presented for comparison in Figure 2. Although CB and CC both increase NR hardness linearly on the basis of their respective loading levels, CB as a reinforcing filler has a more pronounced effect on hardness than the non-reinforcing CC, as is evident in Figure 2a. However, when the plasticizer (PO) is introduced, the hardness is observed to decrease significantly in accordance with the concentration of PO added in Figure 2b,c (for CB) and (for CC). It is interesting that the effect of PO is virtually identical for both CB and CC, even though one is a reinforcing filler and the other is not. The individual effects of these fillers and plasticizing oil on NR hardness agree favorably with those reported elsewhere [13,14,16,17,18]. The hardness prediction from this model can be compared to a previous study [18] investigating the same amount of filler and plasticizer. Both focus on rubbers from the literature and predictions indicate similar hardness levels of 59 and 55, respectively. The difference in hardness might reflect differences in the substances added. However, this model cannot be used to predict hardness when using different types of rubber and additives [2]. While other studies [13,14,16,17] have established the effects of filler and plasticizer on stress-strain behavior relating to hardness, they are unable to compare the value of hardness directly.

**Figure 3:**
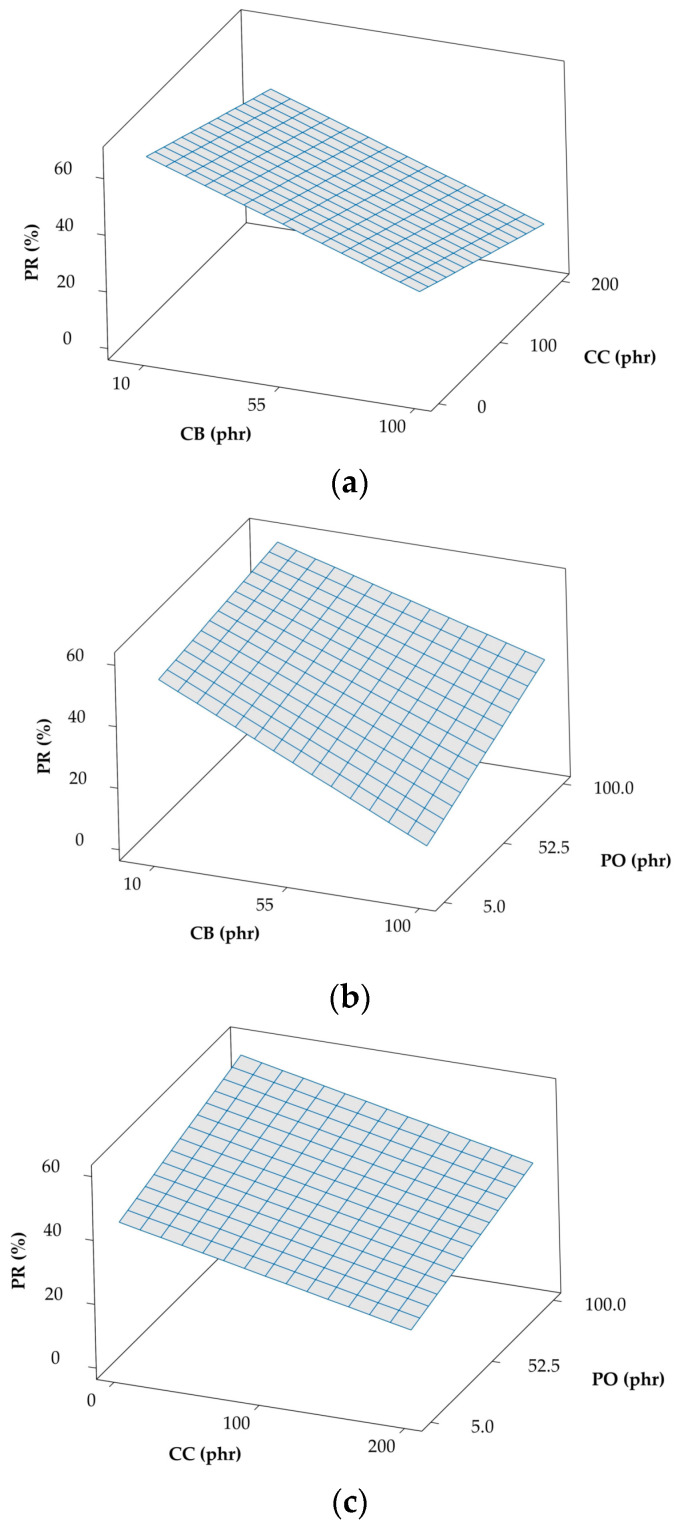
Response cross-correlation surface plots of percentage resilience (PR) with various combinations of additives: (**a**) CC and CB (PO held constant at 52.5), (**b**) PO and CB (CC held constant at 100), and (**c**) PO and CC (CB held constant at 55).

**Figure 4:**
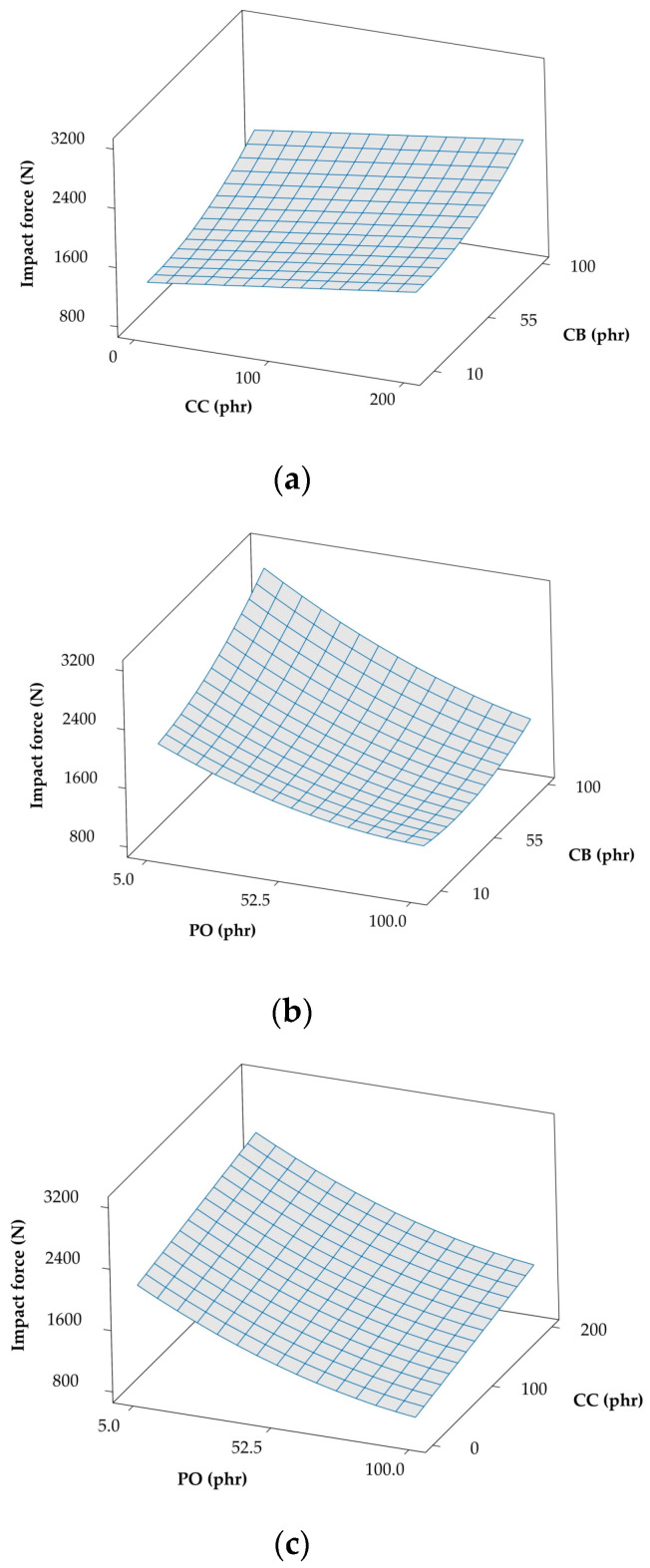
Response cross-correlation surface plots of impact force evaluated at 3 J with various combinations of additives: (**a**) CC and CB (PO held constant at 52.5), (**b**) PO and CB (CC held constant at 100), and (**c**) PO and CC (CB held constant at 55).

Another important property metric, the rebound PR, is commonly employed in testing elastomers to discern the energy lost or dissipated during deformation, since this resilience is inversely proportional to the energy loss. On the basis of the model coefficients that are provided in Table 3, the loading level of CB (X1) is responsible for causing the largest change, a reduction, in PR. The results included in Figure 3a confirm that both CB and CC serve to decrease the PR, most likely by inducing internal friction, which results in energy loss, during deformation. The addition of PO in Figure 3b,c, on the other hand, lessens internal friction, thereby decreasing hysteresis and increasing resilience during deformation. While PR appears from Table 3 to be linear in the loading levels of CB, CC, and PO, the model in Table 3 likewise indicates that a weak correlation exists between CB and CC, although it is not noticeable in Figure 3a. The PR behavior reported here also agrees well with that reported in a previous study [14]. Lastly, according to the requirements for cushioned shoes, materials that transfer a low impact force to the wearer are considered to be preferable for shock absorption. The quadratic regression models in Equation (1) accurately fit experimental data collected at impact energy values ranging from 3 to 7 joules. All of the models of the peak impact force in Table 3 reveal that the term X2 (for PO) significantly reduces the peak impact force. Higher loading levels of fillers (CB and CC) yield higher impact forces that oppose a higher PO content, as displayed in Figure 4. Different loading levels of additives generate the apparent non-linear dependence of the impact force on additive loading levels in this series of plots. In the next section, the model equations presented in Table 3 will be used to optimize the various variables that reduce the force transferred from impact at different impact energy levels.

### 3.3. Impact Force Reduction

**Figure 5:**
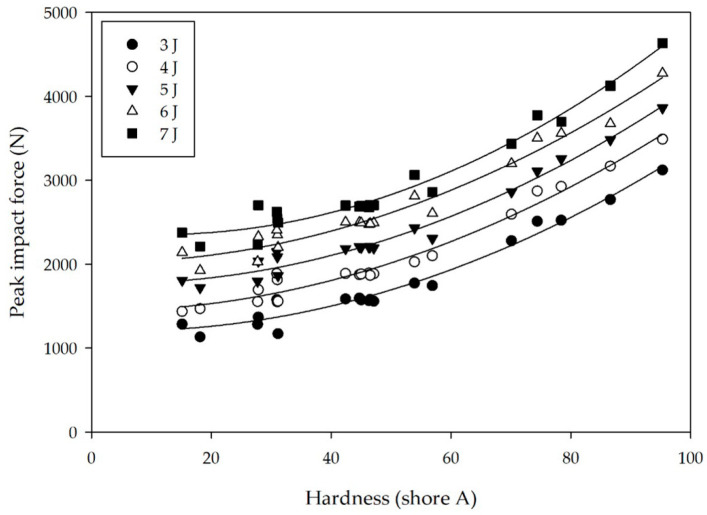
Dependence of peak impact force on natural rubber (NR) hardness evaluated at different energy levels (see legend). Solid lines correspond to quadratic regressions of the data.

Rubber is an example of a viscoelastic material, which can be mathematically represented by a non-linear model that consists of energy-storing elastic springs and energy-dissipating viscous dashpots arranged in a wide variety of combinations (with the two-element serial (Maxwell) and parallel (Kelvin) models representing the most simplistic). In these frameworks, the properties of a viscoelastic material are effectively described by elements corresponding to its stiffness and damping, in which stiffness relates to hardness and damping is related to resilience. Therefore, both of these considerations influence the cushioning property of footwear. In this work, we find that the hardness of modified NR with various concentrations of additives generally follows a polynomial relationship with respect to impact force for each energy level explored, as evidenced by the results that are provided in Figure 5. These findings indicate that NR compounds possessing a low Shore A hardness in the range of 11–31 and formulated with a high PO content are able to substantially reduce the impact force. Conversely, the addition of CB and/or CC tends to increase NR rigidity, thereby leading to an elevated impact force. These results are consistent with those of Silva et al. [2], who have reported that materials with higher energy absorption can absorb greater shock. In that study, energy absorption properties are measured according to a standard test method (EN ISO 20344:2004; Section 5.14) by applying a compressive force up to 5000 N and calculating the energy absorption from the force as a function of displacement. While energy absorption in rubber is related to its hardness with rigid rubber possessing low energy absorption and soft rubber having high energy absorption, Silva et al. [2] report no direct relationship between hardness and impact force reduction. In marked contrast, the present study clearly establishes the existence of such a relationship in Figure 5, namely, rubber with low hardness is able to more effectively reduce impact force than rubber with high hardness. Dura et al. [23] observed that materials exhibiting higher rigidity experience higher impact forces, which agrees favorably with our findings here.

**Figure 6:**
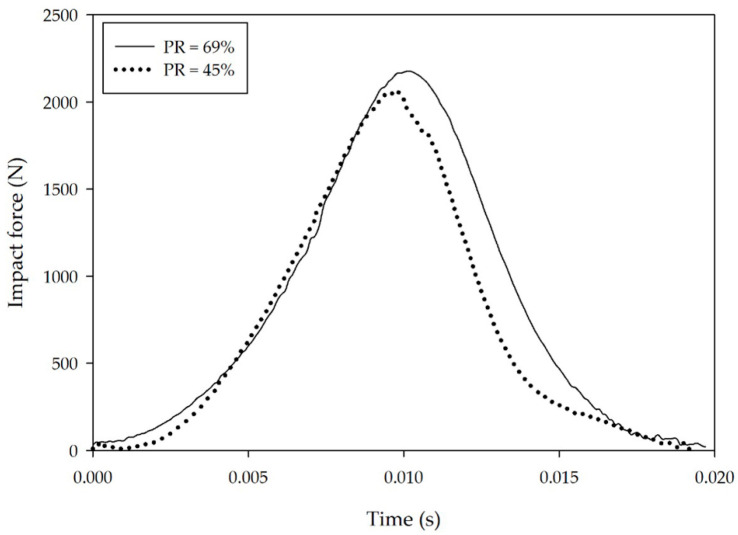
Force-time diagrams of two vulcanized rubbers possessing comparable hardness levels but differing in PR (see legend) evaluated at an impact energy of 6 J. Accompanying slow-motion video reveals that the strain in each case is similar (~60%).

The results of this study do not, however, reveal any direct relationship between PR and impact reduction. Because resilience is the ratio of the energy returned upon recovery from deformation to the energy of deformation, a high PR value translates into a high energy return while a low PR value indicates high energy dissipation. Figure 6 presents the force-time behavior of two rubber compounds under impact loading. They possess similar hardness (18.1 and 15.1 Shore A) but vastly different PR values (45% and 69%, respectively). The results from this test reveal a difference in peak impact force, and the specimen with the low PR value (high damping) displays slightly better impact force attenuation than the one with the high PR value, which is further consistent with the results of Dura et al. [23] and Ramirez and Gupta [24]. Therefore, we conclude that the peak impact force depends on both hardness and PR. Although rubber capable of high damping displays good shock reduction, this property must be considered together with rubber hardness. Increasing the damping in NR can be achieved by increasing the loading level of fillers (CB and CC in this study), but increasing the hardness of the rubber might also adversely affect the impact force. Here, hardness appears to be primarily responsible for impact force attenuation, and so the loading levels of the additives in the formulation are expected to influence not only hardness, but also force attenuation.

### 3.4. Optimal Formulation

**Figure 7:**
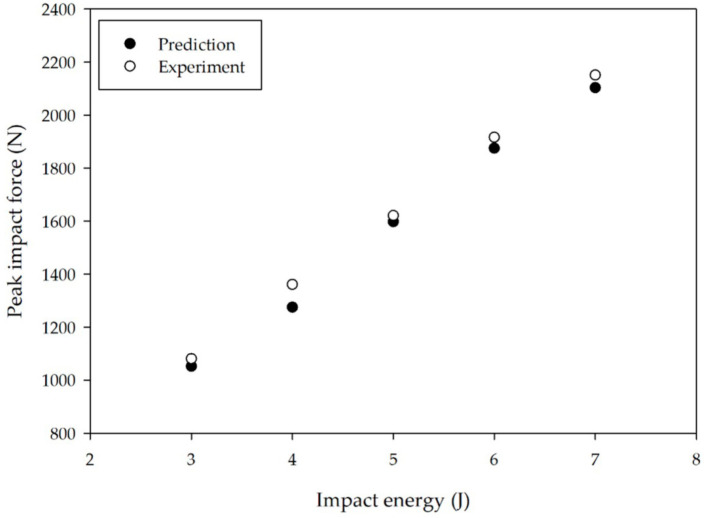
Relationship between peak impact force and impact energy from experimental data and model predictions (see legend).

Because the heel is typically the first part of the foot to contact the ground, it absorbs the brunt of the impact force that is transferred to the leg and body. Therefore, the shock-absorption properties of shoe materials constitute a matter of primary importance in preventing bodily injuries due to incessant impact. The drop-test method adopted in this study provides a systematic means of measuring the shock absorption properties of NR, and the NR compound yielding the lowest peak impact force identifies the best material for shock absorption and, as a consequence, the best material for use in the production of shoe soles. Rubber with a high PR is less effective in reducing the peak impact force than rubber with a low PR, in which case we must also consider energy return as another relevant property. Prior studies [23,25] have examined the benefits of high energy return to reduce oxygen consumption, but materials with high energy loss reduce long-term performance due to energy dissipation as heat by viscous damping [23,25,26,27]. High-resilience materials primarily benefit athletic performance by providing substantial cushioning and energy return. Rubber that is capable of high energy return is associated with high PR values. To determine the optimum loading levels of fillers and plasticizer to achieve the lowest peak impact force with a high PR value, the relationship between the constituents of our NR compounds can be analyzed from the results that are listed in Table 3. Table 4 presents the optimum rubber formulation obtained in this fashion. Although the hardness is quite different between experiment and prediction, it is interesting that (a) PO is the main additive that contributes to NR having both low hardness and high PR and (b) NR with a high PO content is also able to reduce shock effectively. Similar results obtained from experimental data and model predictions are compared in Figure 7. Together, the results reported here confirm that the experiments and predictions are consistent, evincing that this DoE method reliably identifies the most suitable rubber formulation that can be beneficial to the manufacturers of footwear, as well as provides guidance for future research with regard to this subject.

## 4. Conclusions

The main goal of this work is to use the RSM for optimizing the cushioning property of rubber formulations associated with impact force reduction. A DoE study based on the RSM has been performed here to establish the relationships between, as well as the optimum loading levels of, two fillers and a plasticizer in NR compounds used in the manufacture of footwear to reduce the peak impact force and, hence, foot and/or body damage. Experimental data confirms that a large level of plasticizing oil successfully reduces both the peak impact force and the hardness of rubber. On the other hand, both fillers (CB and CC) increase the hardness, thereby leading to a corresponding increase in impact force. High filler levels also promote a low PR (i.e., high energy loss, or dissipation, by the material), whereas the incorporation of the plasticizing oil has the opposite effect. On the basis of the results of this study, impact force attenuation depends on both hardness and the PR, and we have demonstrated that a reduction in impact force is associated with reduced hardness. The RSM greatly facilitates optimizing the response of the rubber compounds studied to changes in composition, and model predictions are consistent with the experimental results. This study has successfully identified the effect of commercially relevant additives in rubber formulations for footwear applications. A predictive RSM model has been developed on the basis of experimental inputs to predict the role of additives (fillers and plasticizers) on mechanical properties (hardness, percentage of elasticity, and maximum impact force), which can be used to discern the most appropriate rubber formulation capable of reducing impact. Our findings verify that quadratic regression models in the context of the RSM yield the optimal loading levels of CB, CC, and PO in NR and predict the properties that correspond to a low impact force. Graphical representations displaying the relationship between impact force reduction and hardness constitute a particularly valuable tool for estimating shock absorption of rubber compounds formulated in the footwear industry. Although hardness is a relatively simple rubber property that can be easily measured, it provides tremendous insight into the ability of NR to absorb impact. Future extensions of this study should focus on other properties, such as the durability of rubber compounds and their resistance to abrasion, friction, tearing, and liquid absorption.

## Figures and Tables

**Table 1:** Rubber compound formulations examined here.

Component	Concentration (phr ^a^)
NR	100
Zinc oxide	3.0
Stearic acid	1.5
Wingstay L ^b^	1.0
MBT	2.0
Sulfur	4.0
CB (X1) ^c^	10 ≤ X_1_ ≤ 100
PO (X2) ^c^	5 ≤ X_2_ ≤ 100
CC (X3) ^c^	0 ≤ X_3_ ≤ 200

^a^ Parts per hundred rubber. ^b^ Antioxidant. ^c^ Independent experimental variables (designation in parentheses).

**Table 2:** Test results based on the design of experiment (DoE) matrix employed in this study.

Specimen	Independent Variables ^a^			Experimental Results						
	X_1_(phr)	X_2_(phr)	X_3_(phr)	Mechanical Properties		Maximum Force at Impact Energy (N)				
				Percentage Resilience (%)	Hardness (Shore A)	3 J	4 J	5 J	6 J	7 J
1	10	100	0	69.10	15.1	1285	1436	1808	2140	2377
2	10	100	200	45.54	18.1	1132	1469	1719	1925	2212
3	10	5	0	62.54	31.0	1551	1814	2087	2350	2540
4	55	52.5	100	44.81	46.3	1565	1893	2204	2475	2681
5	55	52.5	0	47.01	27.7	1283	1554	1796	2030	2234
6	55	52.5	200	30.54	53.9	1773	2026	2432	2810	3063
7	10	5	200	42.65	74.4	2510	2872	3108	3504	3777
8	55	5	100	25.69	78.4	2523	2926	3257	3560	3699
9	100	5	200	9.37	95.3	3122	3490	3864	4276	4635
10	55	52.5	100	43.36	47.1	1558	1885	2193	2493	2703
11	100	100	200	22.29	56.9	1743	2099	2307	2607	2859
12	100	5	0	20.14	86.6	2771	3169	3485	3677	4126
13	55	52.5	100	44.09	46.5	1579	1867	2212	2482	2707
14	10	52.5	100	64.17	30.9	1577	1888	2149	2407	2625
15	55	100	100	45.54	27.8	1368	1695	2035	2328	2702
16	100	52.5	100	21.20	70.1	2279	2595	2862	3201	3437
17	55	52.5	100	45.54	42.4	1585	1888	2186	2501	2699
18	55	52.5	100	45.25	44.7	1595	1874	2209	2498	2688
19	100	100	0	48.50	31.1	1171	1558	1862	2201	2495
20	55	52.5	100	46.27	45.0	1573	1882	2201	2490	2696

^a^ X_1_: carbon black; X_2_: plasticizer oil; X_3_: china clay.

**Table 3:** DoE regressed equations for different testing responses and analysis regression results.

Responses Variable	Model Equations	R^2^	Adjusted R^2^	Predicted R^2^
Hardness (Shore A)	Y = 40.87 + 0.3788X_1_ − 0.4564X_2_ + 0.1072X_3_	0.9051	0.8873	0.8073
Percentage resilience (%)	Y = 63.30 − 0.4588X_1_ + 0.0462X_2_ − 0.09696X_3_ + 0.001861X_1_X_2_	0.9253	0.9054	0.8536
Peak impact force at 3 J (N)	Y = 1893 + 1.89X_1_ − 16.35X_2_ + 2.219X_3_ + 0.0813X_1_^2^ + 0.0807X_2_^2^ − 0.0781X_1_X_2_	0.9219	0.8859	0.6789
Peak impact force at 4 J (N)	Y = 2207 + 2.94X_1_ − 19.18X_2_ + 2.425X_3_ + 0.0767X_1_^2^ + 0.0994X_2_^2^ − 0.0714X_1_X_2_	0.9496	0.9202	0.7713
Peak impact force at 5 J (N)	Y = 2225 + 12.44X_1_ − 19.54X_2_ + 3.834X_3_ + 0.1369X_2_^2^ − 0.08804X_1_X_2_ − 0.0275 X_2_X_3_	0.9419	0.9151	0.7329
Peak impact force at 6 J (N)	Y = 2447 + 12.24X_1_ − 19.31 X_2_ + 4.882 X_3_ + 0.1410X_2_^2^ − 0.0793 X_1_X_2_ − 0.0411X_2_X_3_	0.9434	0.9174	0.7459
Peak impact force at 7 J (N)	Y = 2640 + 14.09 X_1_ − 21.54 X_2_ + 4.911 X_3_ + 0.1724 X_2_^2^− 0.0982X_1_X_2_ − 0.0407X_2_X_3_	0.9394	0.9114	0.7254

**Table 4:** Experimental and predicted optimal compositions of vulcanized NRs properties.

Methodology	CB (phr)	PO (phr)	CC (phr)	PR (%)	Hardness (Shore A)
Predicted	10	82	0	69.10	6.5
Experimental				66.30	16.1
